# Multifunctional NIR-Responsive Composite Hydrogel with Combinatorial Antibacterial and Regenerative Properties for Diabetic Wound Healing

**DOI:** 10.3390/gels12040291

**Published:** 2026-03-31

**Authors:** Shaokai Ji, Chao Wang, Jie Song, Hang Shi, Donglei Wan, Chan Huang, Hanzhi Fu, Xiaohong Cao, Heting Wu, Jian Yang

**Affiliations:** 1 College of Pharmacy, Xinjiang Medical University, Urumqi 830017, China; jisk0994@163.com (S.J.); chaowang9999@outlook.com (C.W.); shi_hang@foxmail.com (H.S.); 19915237276@163.com (D.W.); huang-chan@foxmail.com (C.H.); hanzhi_fu@foxmail.com (H.F.); caoxhong@foxmail.com (X.C.); 2 The First College of Clinical Medicine, Xinjiang Medical University, Urumqi 830017, China; songjiexj2020@163.com; 3 Engineering Research Center of Xinjiang and Central Asian Medicine Resources, Ministry of Education, Urumqi 830011, China

**Keywords:** photothermal composite hydrogel, diabetic chronic wounds, photothermal therapy, antibacterial activity, angiogenesis

## Abstract

The management of diabetic chronic wounds (DFUs) is challenging due to persistent bacterial colonization, impaired neovascularization, and disordered inflammation. We engineered a multifunctional photothermal hydrogel (PPCS) by integrating CuS nanoparticles and high-concentration sucrose to establish a dual-action therapeutic cascade: potent antibacterial eradication followed by pro-angiogenic stimulation. Upon NIR irradiation, the PPCS system executes a combinatorial anti-infective mechanism: CuS-mediated photothermal effect and ROS generation are amplified by sucrose’s hyperosmotic pressure, achieving 99.3% bacterial eradication. Beyond sterilization, the hydrogel acts as a Cu^2+^ sustained-release depot, significantly promoting HUVEC proliferation and migration. This pro-angiogenic effect is mechanistically linked to the upregulation of HIF-1α/VEGF signaling, accelerating neovascularization. Furthermore, sucrose efficiently manages wound exudate, maintaining a repair-conducive microenvironment. In a diabetic rat model, the PPCS dressing demonstrated superior therapeutic efficacy, achieving an accelerated wound closure rate of 99.4% by Day 14, significantly surpassing the control group’s 78.9%. This work presents a tailored hydrogel platform that effectively targets both persistent infection and impaired vascularization, offering a precise and highly efficient therapeutic modality for the clinical management of diabetic chronic wounds.

## 1. Introduction

The effective management of diabetic chronic wounds (DFUs) remains a formidable clinical challenge, primarily due to a complex pathological microenvironment [1,2]. This microenvironment is characterized by several interconnected pathologies: persistent bacterial colonization and biofilm formation, impaired neovascularization, peripheral neuropathy, and a disordered inflammatory cascade that stalls the healing process in the chronic inflammatory phase [3,4]. This pathological state not only precipitates protracted healing but also dramatically amplifies patient morbidity, culminating in a high incidence of non-traumatic lower-limb amputations. The profound socioeconomic burden associated with DFU management underscores the urgent, unmet clinical need for advanced therapeutic strategies that can synergistically target these pathological hallmarks.

While functionalized biomaterials, specifically hydrogels designed for precise drug delivery or growth factor loading, have made notable strides in chronic wound repair, their clinical translation remains severely constrained by deep-seated design challenges. Current strategies predominantly focus on single therapeutic targets, struggling to synchronously modulate the complex and dynamic pathological features of DFUs (e.g., infection, inflammation, and ischemia) in both temporal and spatial dimensions. Firstly, regarding infection control, the pervasive reliance on broad-spectrum antibiotic dressings has irreversibly contributed to a global crisis of antimicrobial resistance (AMR) [5]. Emerging non-antibiotic antimicrobial modalities, such as photothermal therapy (PTT) and photodynamic therapy (PDT), show immense potential in vitro and in acute infection models for effectively disassembling biofilms [6]. However, existing photothermal or photosensitizer carriers are often “monofunctional,” primarily focusing on sterilization but critically lacking the requisite bioactivity or regenerative support needed, resulting in slow wound closure and subpar tissue quality [7]. This “kill-only, rebuild-neglected” strategy fundamentally fails to interrupt the vicious cycle of DFU progression. Secondly, concerning tissue regeneration, while hydrogels loaded with expensive growth factors (e.g., VEGF or PDGF) or stem cells can promote neovascularization, their exorbitant cost, inherent in vivo instability, and rapid enzymatic inactivation within the protease-rich DFU microenvironment limit their sustained therapeutic efficacy [8]. The crux of the challenge lies in material engineering integration. The stable and conflict-free integration of highly efficient, active sterilization functions (e.g., NIR-responsive elements) with mechanisms capable of inducing pro-angiogenesis, immunomodulation, and tissue remodeling (e.g., sustained metal ion release or cytokines) into a single hydrogel matrix remains an unsolved material engineering dilemma. Such integration is a prerequisite for success and must ensure: (1) high dispersibility and photothermal efficiency of nanoparticles (e.g., CuS) during the polymerization process; (2) sustained and effective release kinetics of bioactive components (e.g., Cu^2+^ sources or sucrose); and (3) sufficient mechanical robustness of the final hydrogel for exudate management and adhesion. This profound deficit in Synchronous Multifunctionality significantly curtails the efficacy of existing dressings in rapidly reversing the DFU pathology, underscoring the necessity for developing a next-generation hydrogel platform with active responsiveness, combinatorial antimicrobial action, and robust regenerative capacity.

**Figure 1:**
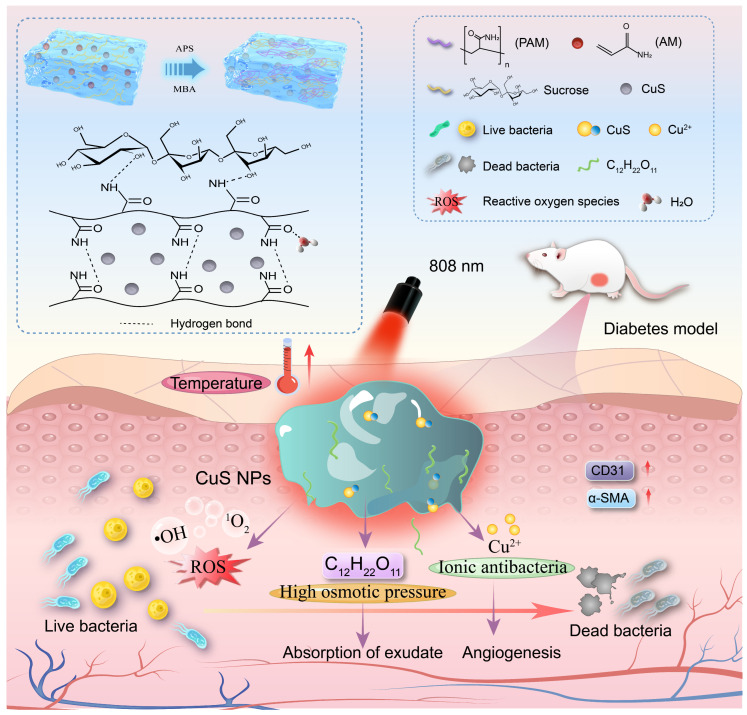
Schematic diagram of hydrogel synthesis and wound healing mechanism.

In this study, we have engineered a tailored photothermal hydrogel (PPCS) based on a polyacrylamide (PAM) backbone, strategically loaded with copper sulfide (CuS) nanoparticles and high-concentration sucrose. This system is designed to execute a combinatorial therapeutic cascade. First, upon 808 nm near-infrared (NIR) irradiation, the CuS NPs exhibit rapid photothermal conversion and reactive oxygen species (ROS) generation, effectively dismantling resilient bacterial biofilms [9,10]. Concurrently, the high-concentration sucrose component exerts significant osmotic pressure, compromising bacterial proliferation [11,12]. This dual-modality approach enables combinatorial antibacterial action, thereby circumventing the pitfalls of conventional AMR. Beyond this potent antibacterial activity, the hydrogel serves as a sustained-release depot for Cu^2+^. This sustained ion release critically promotes neovascularization by upregulating key angiogenic and remodeling markers (CD31 and α-smooth muscle actin, α-SMA), fostering tissue regeneration [13,14]. Furthermore, the hydrogel matrix efficiently manages wound exudate, maintaining a moist microenvironment conducive to repair (Figure 1). Collectively, this work presents a multi-responsive hydrogel platform that integrates photothermal antibacterial therapy with pro-angiogenic stimulation, offering a promising and translatable strategy for the precision management of DFUs.

## 2. Results and Discussion

### 2.1. Synthesis and Characterization of CuS Nanoparticles

**Figure 2:**
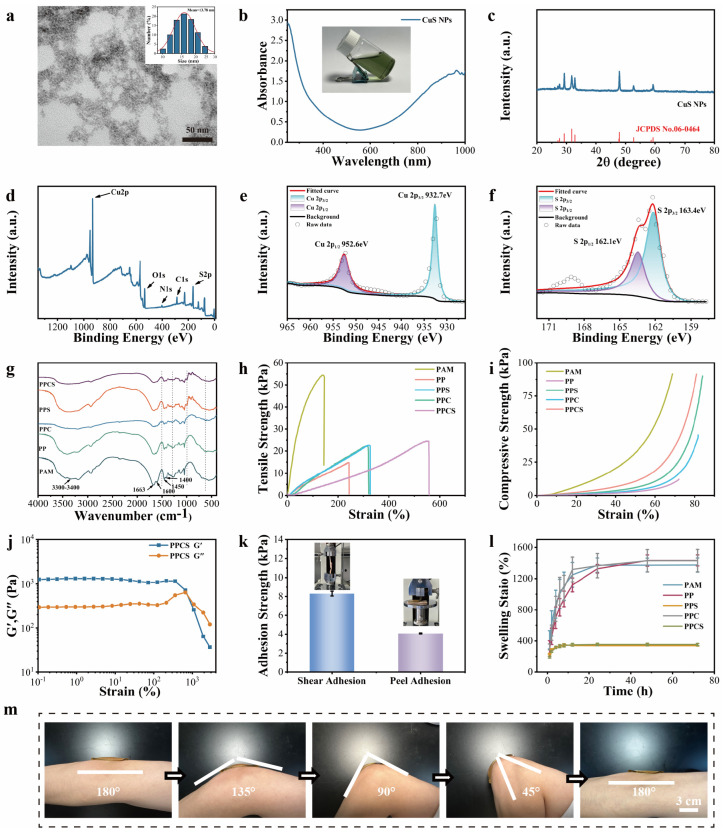
Characterization of CuS NPs and composite hydrogels. (**a**) TEM image and particle size distribution (0–100 nm) of CuS NPs. (**b**) UV-vis absorption spectrum of the CuS NPs dispersion solution. (**c**) XRD pattern of CuS NPs. (**d**) Full XPS spectrum of CuS NPs and (**e**) fine spectra of Cu 2p and (**f**) S 2p. (**g**) Fourier transform infrared (FTIR) spectra of PAM, PP, PPC, PPS, and PPCS. (**h**) Tensile stress–strain curves and (**i**) compressive stress–strain curves of PAM, PP, PPS, PPC, and PPCS hydrogels. (**j**) Changes in G′ and G″ of PPCS hydrogel with increasing strain (strain 1–3000%, frequency 1 Hz). (**k**) Shear adhesion and peel adhesion strength of PPCS hydrogel on pig skin. (**l**) 72 h swelling rates of PAM, PP, PPC, PPS, and PPCS hydrogels (37 °C). (**m**) Adhesion of PPCS hydrogel to the human elbow at various angles.

CuS nanoparticles (CuS NPs) were synthesized via hydrothermal route using CuCl_2_·2H_2_O and Na_2_S·9H_2_O as precursors, with sodium citrate as stabilizer [15]. DLS analysis (0.1 mg/mL) showed average hydrodynamic size of 13.78 nm and PDI of 0.54 (Figure 2a). Zeta potential of −15.77 mV (Figure S1) indicated negatively charged surface with colloidal stability (no sedimentation over 24 h). TEM imaging confirmed spherical morphology (Figure 2a) with slight agglomeration. EDS mapping (Figure S1) showed homogeneous Cu and S distribution. XRD spectra (Figure 2c) displayed characteristic peaks at 2θ = 27.7°, 29.3°, 31.8°, 32.8°, 47.9°, and 52.7°, indexed to hexagonal CuS (JCPDS No. 06-0464). XPS analysis (Figure 2d–f) confirmed binding energy peaks for Cu 2p_3/2_ (932.7 eV), Cu 2p_1/2_ (952.6 eV), S 2p_3/2_ (163.4 eV), and S 2p_1/2_ (162.1 eV), with O and C signals from sodium citrate. UV-Vis-NIR spectroscopy (Figure 2b) demonstrated NIR absorption with absorbance of 1.1 at 808 nm, confirming excellent photothermal conversion potential. The CuS NPs possess uniform size, stable crystal structure, favorable colloidal properties, and strong NIR responsiveness, suitable as photothermal agent for diabetic wound therapy [16].

### 2.2. Preparation and Characterization of PPCS

PPCS composite hydrogels were synthesized via sequential alkali-induced polydopamine (PDA) and free-radical polymerization, alongside PP, PPC, and PPS controls [17]. The uniform yellow-green appearance (Figure S2a) confirmed homogeneous CuS NP dispersion. SEM revealed a highly interconnected 3D porous network (Figure S2b), where sucrose incorporation enlarged pore sizes to enhance exudate absorption [18]. EDS mapping (Figure S2c) further confirmed uniform Cu and S distribution. Chemical integration was validated via FTIR (Figure 2g) and XPS (Figure S3a). FTIR peaks at 619, 991, 1280, and 1480 cm^−1^ corresponded to Cu-S, sucrose C-O, and PDA (quinone and aromatic amine), respectively, indicating a network stabilized by PDA and sucrose interactions. Notably, the sucrose-associated C–O stretching peaks at 991 and 1280 cm^−1^ in the PPCS spectrum exhibit a discernible wavenumber shift and band broadening relative to free sucrose powder, indicative of hydrogen bond formation between the hydroxyl groups (–OH) of sucrose and the amide carbonyl (C=O) and N–H groups of the polyacrylamide network a well-documented FTIR signature of polysaccharide–polyacrylamide hydrogen bonding interactions [19]. XPS further confirmed CuS encapsulation and PDA polymerization. PPCS exhibited robust mechanical properties: 24.49 kPa tensile strength and 556.8% elongation at break (Figure 2h), substantially surpassing those of PAM and single-component formulations. The mechanical superiority of PPCS arises from a hierarchical, multi-mechanism energy dissipation network formed by the synergistic contributions of three structural components. First, PDA formed via alkaline oxidative polymerization of dopamine at pH 11 integrates into the polyacrylamide network through catechol-mediated hydrogen bonding and π–π stacking interactions. These reversible, sacrificial bonds dissipate energy under mechanical deformation without permanent network disruption, accounting for the improvement in mechanical properties from PAM to PP (Figure 2h). Second, the hydroxyl-rich sucrose molecules form extensive hydrogen bonds with the amide groups (C=O and N–H) of polyacrylamide, as evidenced by the FTIR peak shifts at 991 and 1280 cm^−1^ (Figure 2g). These additional sacrificial bond junctions increase the energy required for crack propagation under tensile stress, accounting for the further mechanical improvement from PP to PPS and contributing to the improved compressive modulus (Figure 2i). Third, the negatively charged CuS NPs (zeta potential: −15.77 mV), homogeneously dispersed within the network (Figure S2c), act as physical crosslink nodes through electrostatic and coordination interactions with PDA catechol groups, restricting chain mobility and contributing to tensile strength enhancement from PPS to PPCS. The combination of all three mechanisms creates a hierarchical network with multiple energy dissipation pathways operating at different length scales consistent with toughness-enhancing strategies reported for double-network and nanocomposite hydrogels. Cyclic testing confirmed <10% stress decay after 20 cycles (Figure S4), rheology indicated an exceptional critical strain of 670% (Figure 2j), and immediate self-recovery was demonstrated under various deformations (Figure S5a), ensuring flexibility for joint movements [20,21,22]. Regarding water management, PPCS showed a 353.8% swelling capacity (Figure 2l) and 28.61% water retention over 72 h (Figure S3c). Sucrose and CuS NPs lowered the freezing point to −34.4 °C (Figure S3d) via hydrogen bonding and anti-nucleation effects, enhancing environmental stability. Mussel-inspired PDA integration endowed PPCS with strong tissue adhesion via covalent and hydrogen bonding [23,24]. PPCS achieved shear (8.3 kPa) and peel (4.1 kPa) strengths on pig skin (Figure 2k) while remaining painlessly removable (Figure 2m). These combined properties position PPCS as a promising platform for diabetic wound management, outperforming conventional PAM hydrogels.

### 2.3. Photothermal Performance of PPCS

**Figure 3:**
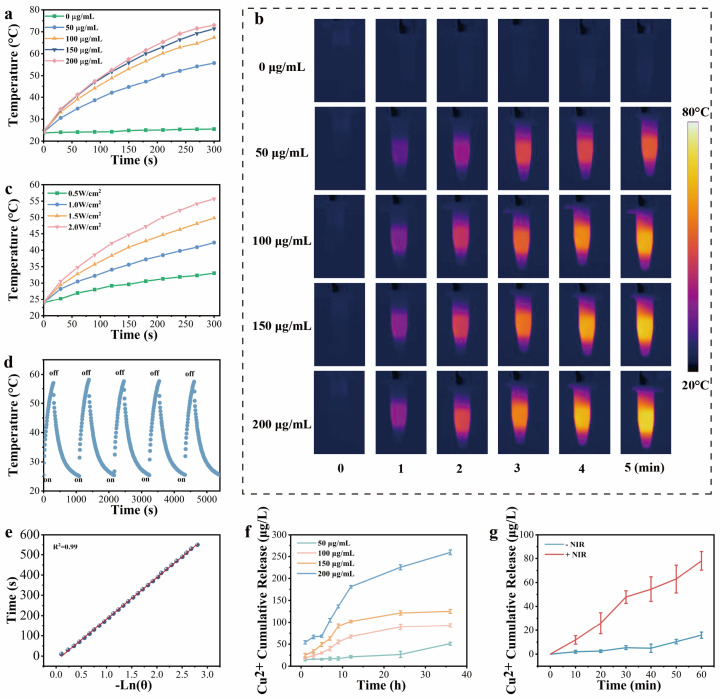
Photothermal performance of PPCS. (**a**) Photothermal heating curves and (**b**) infrared thermal images of PPCS hydrogels with different CuS NPs concentrations under 2 W/cm^2^ 808 nm near-infrared irradiation for 5 min. (**c**) Photothermal heating curves of PPCS hydrogel (CuS NPs 50 μg/mL) at different power densities. (**d**) Temperature change curve of PPCS hydrogel (CuS NPs 50 μg/mL) after 5 on-off cycles under 808 nm near-infrared light at 1.5 W/cm^2^. (**e**) Fitted time constant derived from the relationship between cooling time and the negative natural logarithm of driving temperature during the cooling process. (**f**) Cumulative Cu^2+^ release from PPCS hydrogel in PBS over 36 h at 37 °C (*n* = 3). (**g**) Cumulative Cu^2+^ release from PPCS hydrogel in PBS over 60 min at 37 °C (–NIR and +NIR denote no irradiation and irradiation with 808 nm laser (1.5 W/cm^2^), *n* = 3).

Photothermal properties of PPCS depended on CuS concentration and 808 nm NIR power density (Figure 3a,b). Increasing CuS concentrations (50–200 μg/mL) reached 31.6–48.7 °C after 5 min of 2 W/cm^2^ irradiation, whereas the PP control showed negligible elevation (1.7 °C). To ensure therapeutic safety, 50 μg/mL CuS at 1.5 W/cm^2^ was optimized to reach 49.8 °C (Figure 3c), providing bactericidal action without tissue damage. PPCS demonstrated high photothermal stability over 5 cycles (Figure 3d) and a 56.5% conversion efficiency (Figure 3e).

Furthermore, NIR irradiation significantly accelerated Cu^2+^ release compared to the sustained baseline release observed over 36 h (Figure 3f,g). This on-demand release, driven by heat-enhanced molecular diffusion [25], combined with optimized photothermal performance, makes PPCS a promising multi-functional platform for diabetic wound management [26].

### 2.4. In Vitro Biocompatibility and Hemocompatibility Assessment of PPCS

**Figure 4:**
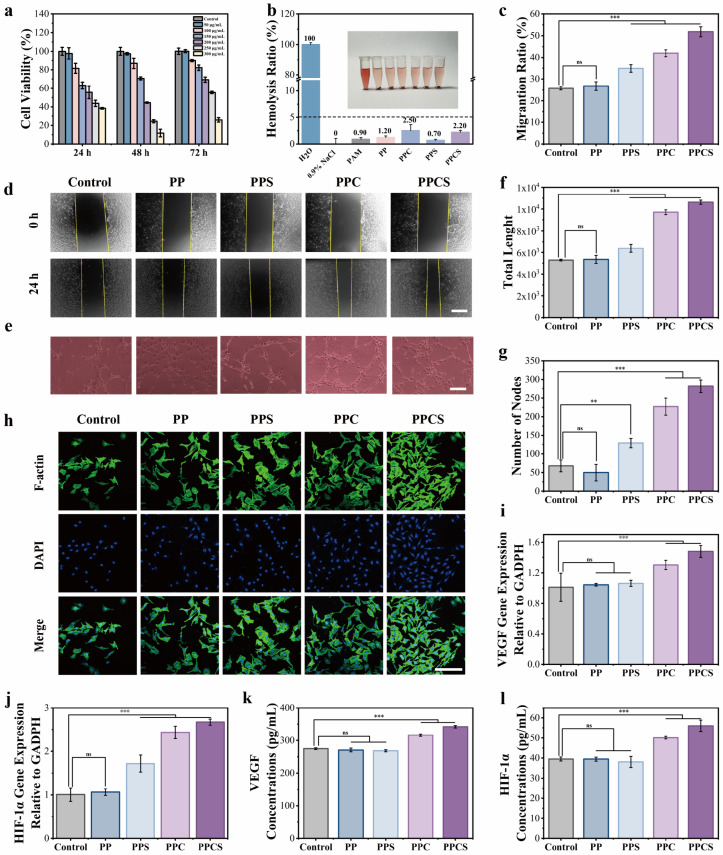
In vitro biocompatibility and cellular functionality tests of hydrogels. (**a**) Cell viability of HUVECs co-cultured with PPCS hydrogel extracts at different CuS NPs concentrations for 24, 48, and 72 h (*n* = 3). (**b**) Hemolysis rates of H_2_O (positive control), 0.9% NaCl (negative control), PAM, PP, PPC, PPS, and PPCS hydrogels (*n* = 3). (**d**) Images of scratch healing after 24 h treatment of HUVECs with different hydrogel extracts and (**c**) cell migration rate (*n* = 3, scale bar: 400 μm). (**e**) Matrigel-based in vitro tube formation of HUVECs (scale bar: 400 μm). (**f**) Image J quantification of total length and (**g**) Image J quantification of tube junctions (*n* = 3). (**h**) Phalloidin staining images of HUVECs co-cultured with PBS (control), PP, PPS, PPC, and PPCS hydrogel extracts for 3 days; F-actin stained green, nuclei stained blue (scale bar: 200 μm). (**i**) RT-qPCR detection of VEGF and (**j**) HIF-1α levels in cell supernatants after 3 days of co-culture between HUVECs and different hydrogel extracts (*n* = 3). (**k**) ELISA detection of VEGF and (**l**) HIF-1α levels in cell supernatants after co-culturing HUVECs with different hydrogel extracts for 3 days (*n* = 3). (** *p* < 0.01, *** *p* < 0.001, ns indicates not statistically significant).

Biocompatibility of PPCS was evaluated using HUVECs and L929 fibroblasts. CCK-8 assays demonstrated that the optimized PPCS (50 μg/mL CuS, 72 h maintained 100.2 ± 1.7% HUVEC viability, comparable to the control (*p* > 0.05, Figure 4a). Across 24–72 h, all formulations (PP, PPC, PPS, PPCS) exhibited viability >90% (Figure S6), confirming broad cytocompatibility. Live/dead staining (Figure S7) corroborated low cytotoxicity, with PPC and PPCS groups showing enhanced HUVEC proliferation, likely due to pro-angiogenic low-dose Cu^2+^ [27].

FITC-phalloidin staining (Figure 4h) showed that PPCS-treated cells maintained characteristic polygonal morphology with intact F-actin stress fibers and increased cellular density compared to controls, suggesting a microenvironment conducive to wound repair [28].

The hemocompatibility of all hydrogel formulations was assessed via a standard hemolysis assay. As shown in Figure 4b, the hemolysis rates of PAM, PP, PPC, PPS, and PPCS were 0.9 ± 0.3%, 1.2 ± 0.3%, 2.5 ± 1.1%, 0.7 ± 0.2%, and 2.2 ± 0.3%, respectively, all well below the internationally accepted biosafety threshold of 5% (ASTM F756/ISO 10993-4) [29,30], confirming excellent hemocompatibility across all formulations. Notably, the slightly elevated hemolysis rates observed in CuS-containing groups (PPC: 2.5%; PPCS: 2.2%) relative to the Cu-free controls remain within the safe range and are attributable to the trace release of Cu^2+^ ions, which at low concentrations may interact with erythrocyte membranes without causing clinically meaningful hemolytic damage. The sucrose-containing PPS group exhibited the lowest hemolysis rate (0.7%), consistent with the osmotic-stabilizing effect of sucrose on red blood cell membranes under the assay conditions. These results collectively confirm that the PPCS hydrogel platform is hemocompatible and safe for application as a blood-contacting wound dressing.

### 2.5. Pro-Angiogenic Capacity and Mechanistic Validation via Cu^2+^-Mediated HIF-1α/VEGF Pathway Upregulation

The pro-angiogenic capacity of PPCS was evaluated using HUVECs. Scratch assays (Figure 4c,d) revealed 24-h migration rates of 26.8% (PP), 34.9% (PPS), 42.0% (PPC), and 51.8% (PPCS), all significantly higher than the control (25.8%). Tube formation assays (Figure 4e,f,i) further showed that PPCS doubled total tube length and increased node numbers by ~315%. This enhanced performance in PPC and PPCS groups correlates with Cu^2+^ release, a key factor for vascular regeneration.

RT-qPCR (Figure 4i,j) and ELISA (Figure 4k,l) demonstrated significantly elevated HIF-1α and VEGF expression at both the mRNA and protein levels in the PPC and PPCS groups (*p* < 0.001), whereas the Cu-free PP group sharing an identical polyacrylamide/polydopamine backbone but releasing no Cu^2+^ showed no significant upregulation compared to the PBS control (*p* > 0.05). The PP group showed significant differences compared to the PPC and PPCS groups (*p* < 0.001). This systematic contrast between Cu^2+^-releasing groups (PPC, PPCS) and the matched Cu-free control (PP) directly implicates sustained Cu^2+^ release, rather than the polymer matrix or other hydrogel components, as the causal mediator of HIF-1α/VEGF pathway activation.

Mechanistically, this Cu^2+^-driven normoxic HIF-1α stabilization proceeds through a well-characterized pathway: exogenous Cu^2+^ ions suppress the activity of prolyl hydroxylase domain (PHD) enzymes, which under normal oxygen tension continuously hydroxylate HIF-1α proline residues and target the protein for proteasomal degradation via the von Hippel–Lindau (VHL) E3 ubiquitin ligase complex. By chelating the Fe^2+^ cofactor required for PHD catalytic activity, Cu^2+^ disrupts this oxygen-sensing degradation axis, allowing HIF-1α to accumulate, translocate to the nucleus, and drive transcription of downstream targets most critically VEGF the primary paracrine driver of endothelial proliferation, migration, and tube formation [31]. This mechanism has been explicitly demonstrated in copper-doped biomaterial systems and is consistent with the dose-dependent pro-angiogenic responses observed in our scratch assay and tube formation data (Figure 4c–g) [32,33]. Collectively, these findings establish that PPCS promotes neovascularization in diabetic wounds through a Cu^2+^-specific, PHD-mediated stabilization of the HIF-1α/VEGF signaling axis, providing a rigorous mechanistic foundation for its pro-angiogenic efficacy and translational repair potential.

### 2.6. In Vitro Antibacterial Performance of PPCS

**Figure 5:**
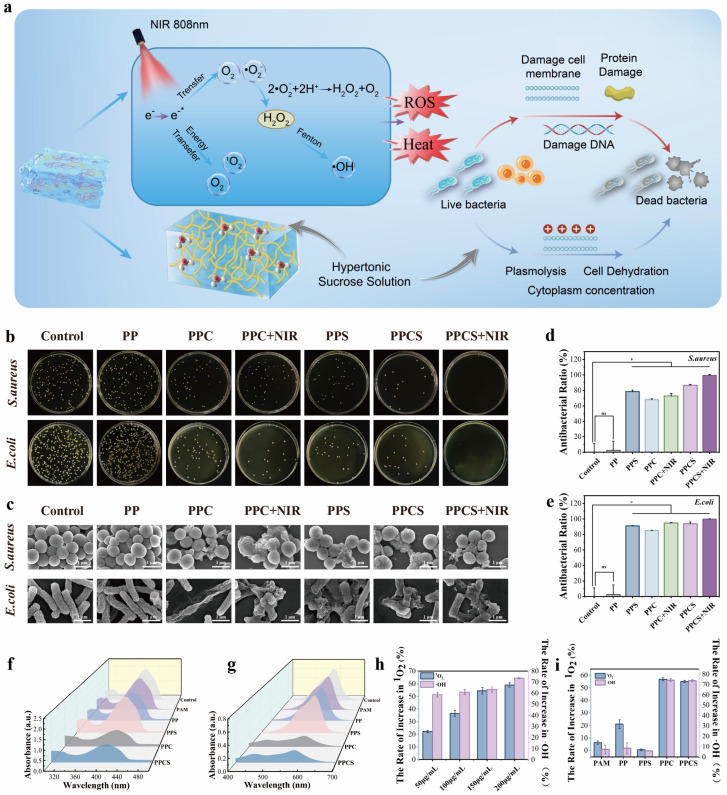
Antibacterial activity and in vitro ROS generation of different hydrogels. (**a**) Schematic illustration of the antimicrobial mechanism of PPCS hydrogel. (**b**) Photographs of bacterial plate counts after co-culturing each hydrogel group with *S. aureus* and *E. coli* for 4 h. (**c**) SEM images of *S. aureus* and *E. coli* after treatment with different hydrogels (scale bar: 1 μm). (**d**) Antibacterial ratio of *S. aureus* and (**e**) *E. coli* after treatment with different hydrogels (*n* = 3). (**f**) UV absorption spectra of different hydrogels at 420 nm and (**g**) 580 nm. (**h**) Hydroxyl radical (·OH) and singlet oxygen (^1^O_2_) generation rates of PPCS hydrogels with different CuS NPs concentrations after 10 min NIR irradiation. (**i**) Hydroxyl radical (·OH) and singlet oxygen (^1^O_2_) generation rates of different hydrogels after 10 min NIR irradiation. (*n* = 3). (* *p* < 0.05, ns indicates not statistically significant).

PPCS executes a multi-modal antibacterial strategy (Figure 5a). Under NIR irradiation, CuS NPs generate electron-hole pairs, producing reactive oxygen species (ROS) such as ^1^O_2_ and ·OH that cause irreversible bacterial damage [34,35]. This effect is amplified by photothermal hyperthermia, sucrose-induced hyperosmotic stress (plasmolysis), and sustained Cu^2+^ release. This multi-pronged approach mitigates bacterial resistance compared to single-mode treatments [36]. Quantitative plate counting (Figure 5b) showed that PPCS + NIR achieved near-complete eradication (≈100%) of *E. coli* and *S. aureus* (*p* < 0.05). The additive multi-modal contribution was evident from systematic group comparison: the PPC group (CuS NPs, no NIR) achieved 68.1% (*S. aureus*) and 85.1% (*E. coli*) killing through Cu^2+^ release and residual dark-state ROS activity, representing the baseline without photothermal or NIR-induced ROS contributions; the PPS group (sucrose, no CuS, no NIR) achieved 78.6% (*S. aureus*) and 91.1% (*E. coli*) killing via hyperosmotic stress alone; and the PPCS group (CuS + sucrose, no NIR) achieved intermediate killing through the combined Cu^2+^ and osmotic mechanisms. The incremental contribution of NIR irradiation encompassing both photothermal hyperthermia and NIR-induced ROS is reflected in the step increase from PPCS to PPCS + NIR (≥99.3%), confirming that NIR activation is indispensable for near-complete eradication. SEM imaging (Figure 5c) confirmed morphological destruction consistent with both thermal and oxidative damage, including cell wall perforation, membrane rupture, and intracellular content leakage.

ROS generation was quantified via UV-visible spectrophotometry (Figure 5f–i), showing generation rates of 73.7% (·OH) and 55.1% (^1^O_2_). This high photocatalytic activity results from NIR-induced electron-hole pair formation on CuS. Collectively, the PPCS + NIR system provides superior anti-infective capacity for diabetic wound management, Superior to some antibiotics currently in clinical use (Table S3). Analogous multi-modal photothermal/ROS platforms have been explicitly demonstrated to retain near-complete killing efficacy against MRSA and other multidrug-resistant (MDR) clinical isolates in multiple recent studies [37,38].

### 2.7. In Vivo Evaluation of PPCS: Accelerated Diabetic Wound Regeneration

**Figure 6:**
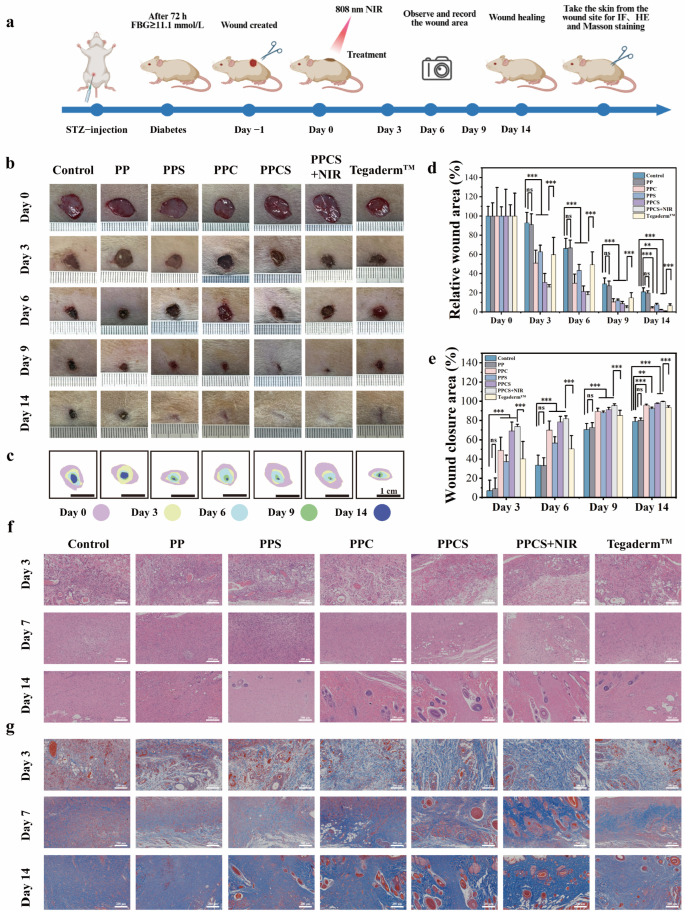
Wound healing promotion and histopathological analysis of diabetic rat wounds treated with different hydrogels. (**a**) Schematic diagram of diabetic rat wound model establishment and hydrogel treatment process. (Created with BioRender.com) (**b**) Wound images of diabetic rats treated with different hydrogels at days 0, 3, 6, 9, and 14. (**c**) Schematic diagram of the wound healing process (scale bar: 1 cm). (**d**) Quantitative analysis of relative wound area at days 0, 3, 6, 9, and 14 after wound treatment with different hydrogels. (**e**) Quantitative analysis of wound healing area at days 0, 3, 6, 9, and 14 after wound treatment with different hydrogels (*n* = 6). (**f**) H&E staining of wound skin tissue at days 3, 7, and 14 after treatment with different hydrogels. (**g**) Masson’s trichrome staining of wound skin tissue at days 3, 7, and 14 after treatment with different hydrogels (scale bar: 200 μm). (** *p* <0.01, *** *p* < 0.001, ns indicates not statistically significant).

The therapeutic efficacy of PPCS + NIR was evaluated using a diabetic rat dorsal skin defect model across seven groups, including Tegaderm™ as a positive control. Wound area analysis (Figure 6a–c) showed that PPCS + NIR achieved the most rapid closure, reaching a near-complete healing rate of 99.4 ± 0.4% by Day 14. This significantly outperformed PPS (92.4%), PPC (95.8%), PPCS (97.7%), and Tegaderm™ (93.4%) (*p* < 0.05, Figure 6d,e).

H&E and Masson staining (Figure 6f,g) characterized the tissue regeneration quality. By Day 3, PPCS and PPCS + NIR groups showed markedly reduced inflammatory cell infiltration. By Day 7, these groups successfully transitioned out of the chronic inflammatory phase. By Day 14, PPCS + NIR exhibited the most mature tissue, featuring robust granulation, neodermis formation, and regenerated dermal appendages (hair follicles and sebaceous glands). Masson’s trichrome staining confirmed that PPCS + NIR induced the most extensive collagen deposition and ECM remodeling throughout the 14-day period.

**Figure 7:**
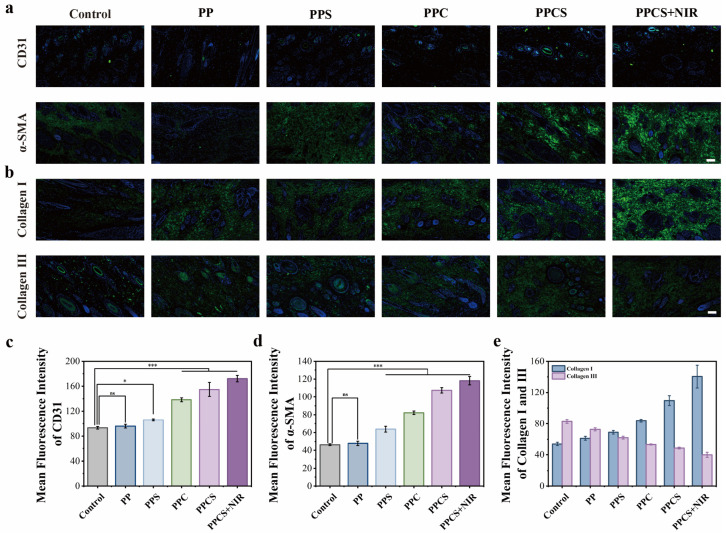
Hydrogel promotes angiogenesis and collagen remodeling in diabetic wounds by upregulating angiogenesis-related growth factors and collagen. (**a**) Immunofluorescence staining images of regenerated skin tissue at day 14 of PPCS hydrogel treatment showing CD31 (green) and α-SMA (green) (scale bar: 100 μm). (**b**) Immunofluorescence staining images of regenerated skin tissue treated with PPCS hydrogel at day 14, showing Collagen I (green) and Collagen III (green) (scale bar: 100 μm). (**c**) Statistics of average fluorescence intensity for (**c**) CD31, (**d**) α-SMA, (**e**) Collagen I, and Collagen III (*n* = 3). Green fluorescence represents the respective markers, while blue fluorescence indicates cell nuclei. (* *p* <0.05, *** *p* <0.001, ns indicates not statistically significant).

It should be noted that the STZ-induced diabetic rat dorsal wound model employed in this study was conducted under aseptic surgical conditions without exogenous bacterial inoculation, and therefore represents a sterile wound model. The absence of overt infection in the control group is consistent with this design, and the observed in vivo therapeutic superiority of PPCS + NIR over control groups is primarily attributable to its pro-angiogenic (Cu^2+^–HIF-1α/VEGF axis activation), anti-inflammatory, and ECM remodeling activities, as evidenced by the histological and immunofluorescence data (Figure 6f,g and Figure 7), rather than to its antibacterial mechanisms per se. The antibacterial efficacy of PPCS + NIR is independently established through the dedicated in vitro experiments described in Section 2.6 (Figure 5), where near-complete eradication (≥99.3%) of both *S. aureus* and *E. coli* was unambiguously demonstrated.

In conclusion, the PPCS + NIR system significantly accelerates diabetic wound healing through a combinatorial cascade: mitigating chronic inflammation, promoting granulation, and enhancing collagen-driven ECM remodeling. This superior performance, driven by NIR-induced ROS, photothermal effects, and Cu^2+^/sucrose activity, positions PPCS as a highly translatable platform for clinical diabetic wound management. While the final wound closure rates of PPCS (97.7%) and PPCS + NIR (99.4%) at Day 14 appear numerically proximate, a temporally resolved analysis reveals a more substantive contribution of NIR irradiation during the critical early healing phases. As shown in Figure 6d,e, the PPCS + NIR group demonstrated consistently faster wound closure compared to PPCS at Days 3, 6, and 9, reflecting the role of NIR-triggered photothermal hyperthermia in two early-phase functions: rapid bactericidal action that prevents the establishment of subclinical infection, and thermal vasodilation that transiently enhances local perfusion and accelerates the transition from the chronic inflammatory phase to the proliferative phase. This temporal advantage is corroborated by histological analysis (Figure 6f,g): at Day 3, the PPCS + NIR group exhibited markedly reduced inflammatory cell infiltration relative to PPCS, and by Day 7, PPCS + NIR had more completely resolved the chronic inflammatory state. By Day 14, while both groups achieved high closure rates, PPCS + NIR produced demonstrably superior tissue architecture including more mature granulation tissue, denser and more organized collagen deposition (Masson staining, Figure 6g), indicating that the NIR contribution extends beyond wound area reduction to encompass the quality and maturity of regenerated tissue. Collectively, these data establish that NIR irradiation provides distinct and measurable therapeutic value throughout the healing timeline, with its greatest impact concentrated in the early inflammatory-to-proliferative transition window.

### 2.8. Comprehensive Analysis of Angiogenesis and ECM Remodeling in Regenerated Diabetic Tissue

Neovascularization and collagen remodeling are pivotal determinants of successful wound healing, with the former supplying essential nutrients for the proliferative phase and the latter governing regenerative healing quality by achieving organized collagen deposition [39]. Neovascularization and collagen remodeling were assessed via immunofluorescence staining for CD31, α-SMA, and Collagen I/III. Quantitative analysis (Figure 7a,c,d) showed significantly higher vessel density in PPC, PPCS, and PPCS + NIR groups than the control (*p* < 0.001). The PPCS + NIR group achieved maximal CD31 and α-SMA expression, confirming robust angiogenesis and vascular maturation driven by Cu^2+^-activated HIF-1α signaling and NIR-induced thermal vasodilation.

Collagen analysis (Figure 7b,e) revealed that while other groups maintained high Collagen III/I ratios indicative of immature scarring, PPCS + NIR exhibited dense, organized fibers with a Collagen I/III ratio (≈3–4:1) resembling native skin. This indicates an accelerated transition from Collagen III to mechanically superior Collagen I, suggesting high-quality regenerative healing. Such superior ECM remodeling stems from the platform’s integrated antimicrobial, anti-inflammatory, and NIR-induced stimulatory effects. Future studies will validate its long-term stability and broader clinical applicability.

### 2.9. In Vivo Safety Evaluation

The in vivo safety of PPCS was evaluated via H&E staining of major organs (heart, liver, spleen, lung, and kidney) after 14 days. Both PPCS and PPCS + NIR groups exhibited intact tissue morphology without inflammatory infiltration, fibrosis, or necrosis (Figure S8), confirming excellent biocompatibility attributed to the controlled Cu^2+^ release and intrinsic safety of CuS NPs.

The superior efficacy of PPCS + NIR stems from its combinatorial multi-mechanism action: (1) a triple-action antibacterial strategy combining sucrose-induced hyperosmotic stress, NIR-triggered ROS generation, and photothermal hyperthermia; (2) rapid mitigation of chronic inflammation; and (3) sustained Cu^2+^ release that activates the HIF-1α/VEGF pathway to promote angiogenesis, granulation tissue formation, and Collagen I remodeling.

In summary, the PPCS + NIR hydrogel provides a safe and effective whole-process management platform for chronic diabetic wounds, offering high clinical translatability for accelerated regenerative healing.

## 3. Conclusions

Diabetic foot ulcers present a dual clinical challenge: persistent infection that perpetuates chronic inflammation, and impaired neovascularization that prevents tissue regeneration. This study engineered the PPCS photothermal composite hydrogel to simultaneously resolve both barriers through a single integrated platform.

For infection control, PPCS + NIR achieved near-complete eradication of *S. aureus* and *E. coli* (≥99.3%) via three concurrent physical mechanisms NIR-triggered photothermal hyperthermia (≥49.8 °C), ROS generation (·OH: 73.7%; ^1^O_2_: 55.1%), and sucrose-induced hyperosmotic stress circumventing conventional antibiotic resistance.

For tissue regeneration, sustained Cu^2+^ release drove pro-angiogenic activity: HUVEC migration reached 51.8% versus 25.8% in controls, with tube length doubled and node numbers increased by ~315%. Mechanistically, Cu^2+^ stabilized HIF-1α via PHD enzyme inhibition, significantly elevating HIF-1α and VEGF expression in Cu^2+^-releasing groups relative to the matched Cu-free PP control (*p* < 0.001), while PP showed no upregulation over PBS (*p* > 0.05). In the diabetic rat model, PPCS + NIR achieved 99.4 ± 0.4% wound closure by Day 14, surpassing both the untreated control (78.9%) and Tegaderm™ (93.4%) (*p* < 0.05). Histological analysis confirmed robust neovascularization (CD31/α-SMA upregulation), rapid inflammatory resolution, and mature ECM remodeling with a Collagen I/III ratio of ~3–4:1 approaching native skin. Systemic safety was confirmed by intact major organ morphology at Day 14.

Collectively, PPCS + NIR demonstrates that a sequential therapeutic cascade infection eradication followed by pro-angiogenic stimulation can be encoded into a single hydrogel through rational integration of CuS nanoparticles, sucrose, and a polydopamine-reinforced polyacrylamide backbone, offering a mechanistically grounded and clinically translatable platform for chronic diabetic wound management.

Future work will focus on four directions: validating antibacterial efficacy against MRSA and MDR clinical isolates using a bacterially inoculated wound model; evaluating cellular responses under mild hyperthermic conditions (42–45 °C) to assess HSP-mediated pro-regenerative benefits; characterizing long-term sucrose retention kinetics and mechanical stability; and conducting large-animal model validation toward clinical translation.

## 4. Materials and Methods

### 4.1. Materials

CuCl_2_ (Damao, Tianjin, China), Na_2_S·9H_2_O, and sodium citrate were purchased from Zhiyuan (Tianjin, China). Acrylamide (AM), N,N-Methylenebisacrylamide (MBA), ammonium persulfate (APS), dopamine hydrochloride, and sucrose (99%) were purchased from Macklin (Shanghai, China). HUVECs and L929 cells were obtained from the Chinese Academy of Sciences Cell Bank (Shanghai, China). Fetal bovine serum, 0.25% trypsin digestion solution, CCK-8 cell proliferation and toxicity assay kit, and AF488 phalloidin were purchased from Heyuan Li Ji Biological (Shanghai, China). DMEM high-glucose medium (Gibco, Carlsbad, CA, USA), Calcein-AM/PI dual-staining kit for live/dead cells (Solaibao, Beijing, China), and Matrigel matrix gel (Corning, New York, NY, USA). Highly specific qPCR reagent TB Green and quantitative PCR-specific reverse transcription kit were purchased from Takara (Beijing, China). Vascular Endothelial Growth Factor (VEGF) and Hypoxia-Inducible Factor 1α (HIF-1α) ELISA kits were purchased from Lianke Bio (Hangzhou, China). *E. coli* (ATCC 25922) and *S. aureus* (ATCC 29213) were purchased from Wanjia Shouhua Biotechnology Co., Ltd. (Beijing, China). Streptozotocin (STZ) was purchased from Boaigang (Beijing, China). Tegaderm™ (3M, St. Paul, MN, USA No. 1626W). Collagen I and Collagen III antibodies were obtained from Abcam (Cambridge, UK). CD31 antibody was purchased from CST (Danvers, MA, USA). α-SMA antibody was acquired from Wuhan Sanying (Wuhan, China). CY3-labeled goat anti-rabbit IgG was sourced from Bioss (Beijing, China). SD rats (6–8 weeks old, male) were purchased from the Animal Experiment Center of Xinjiang Medical University (Urumqi, China). The animal experiment protocol and procedures were approved by the Animal Research and Ethics Committee of Xinjiang Medical University (Ethics Approval No.IACUC-JT-20250311-06).

### 4.2. Preparation and Characterization of CuS NPs

CuS nanoparticles were synthesized via a hydrothermal method. Briefly, CuCl_2_·2H_2_O (17 mg, 0.1 mmol) and sodium citrate (20 mg, 0.068 mmol) were dissolved in 100 mL of ultrapure water under magnetic stirring at room temperature until a homogeneous transparent solution was obtained. Na_2_S·9H_2_O (24 mg, 0.1 mmol) was separately dissolved in 1 mL of ultrapure water and added dropwise to the CuCl_2_/sodium citrate solution under continuous stirring at a rate of approximately 1 mL/min. The molar ratio of Cu^2+^ to S^2−^ was maintained at 1:1, with sodium citrate serving as a surface stabilizer at a citrate: Cu^2+^ molar ratio of approximately 0.68:1. The reaction was conducted without pH adjustment; the as-prepared mixture exhibited a near-neutral pH of 6.8–7.2 as measured by calibrated pH meter prior to heating. The mixture was stirred for 5 min at room temperature to allow initial nucleation, then transferred to a pre-heated water bath and reacted at 90 °C for 15 min under continuous magnetic stirring. The resulting dispersion was cooled naturally to room temperature, then filtered through a 0.22 μm polyethersulfone (PES) membrane to remove any large aggregates. The purified CuS NP dispersion was stored at 4 °C protected from light prior to use. The as-prepared CuS NPs were characterized by transmission electron microscopy (TEM, H7800, Hitachi Limited, Tokyo, Japan), dynamic light scattering and zeta potential analysis (Malvern Zetasizer Nano ZS, Malvern Instruments Ltd., Worcestershire, UK), X-ray diffraction (XRD, Malvern PANalytical, Malvern Instruments Ltd., Worcestershire, UK), X-ray photoelectron spectroscopy (XPS, Thermo Scientific K-Alpha, Thermo Fisher Scientific, Wilmington, DE, USA), and UV-vis-NIRspectroscopy (HORIBA Duetta spectrometer, Kyoto, Japan).

### 4.3. Preparation and Characterization of Hydrogels

Dissolve 0.0125 g of dopamine hydrochloride in 5 mL of NaOH solution (pH = 11) and stir for 20 min in air to form polydopamine (PDA). Dissolve 2.5 g AM, 0.025 g MBA, and 0.25 g APS in 10 mL deionized water. Combine with the PDA solution and polymerize under vacuum drying at 60 °C for 2 h to form PP hydrogel. PPC hydrogel: Add CuS NPs (concentration 50–200 μg/mL) prior to polymerization, stir thoroughly in an ice bath, and polymerize at 60 °C for 2 h. PPS hydrogel: Add 4.5 g sucrose (30% *w*/*v*) prior to polymerization and polymerize as above. PPCS hydrogel: Add CuS NPs (50–200 μg/mL) and 4.5 g sucrose prior to polymerization, stir on ice bath, and polymerize at 60 °C for 2 h. Freeze-dried PPCS hydrogel cross-section porous structure observed by scanning electron microscopy (SEM, ZEISS Sigma 360, Carl Zeiss AG, Oberkochen, Germany) after gold spraying. Freeze-dried samples were pressed into KBr pellets and analyzed for functional groups using Fourier transform infrared spectroscopy (FTIR, SHIMADZU IR Trace-100, SHIMADZU Corporation, Kyoto, Japan). XRD (XRDynamic 500, The Anton Paar Group, Graz, Austria) characterized the crystal structures of CuS NPs and PPCS. Differential scanning calorimetry (DSC, Netzsch DSC 200 F3, Netzsch-Gerätebau GmbH, selb, Germany) assessed freeze resistance from −80 °C to 20 °C. A rotational rheometer (Malvern KNX2110, Malvern Instruments Ltd. Worcestershire, UK) was used to measure the storage modulus (G′) and loss modulus (G″) at 25 °C through frequency scanning (0.01–10 Hz) and strain scanning (1–1000%).

### 4.4. Hydrogel Performance Testing

(1) Adhesion: Shear and peel adhesion tests were performed on fresh porcine skin. The skin surface was gently blotted with absorbent paper to remove excess surface moisture while retaining its native hydrated state, simulating moist wound contact conditions. For shear adhesion testing, hydrogel specimens (20 mm × 20 mm × 2 mm) were placed on the porcine skin surface; for 180° peel adhesion testing, specimens of 20 mm × 80 mm × 2 mm were used. A uniform contact pressure of approximately 10 kPa was applied by placing a 500 g dead weight on the hydrogel for a contact time of 60 s prior to mechanical testing. All tests were conducted at room temperature (25 ± 2 °C) and ambient humidity (50 ± 5% RH) using a universal mechanical testing machine at a crosshead speed of 10 mm/min for shear and 25 mm/min for 180° peel. Shear adhesion strength was calculated as the maximum shear force divided by the bonded contact area (kPa), and peel adhesion strength was reported as force per unit width (N/m). All measurements were performed in triplicate (*n* = 3).

(2) Swelling: Hydrogels immersed in PBS (pH 7.4, 37 °C), weighed at intervals (*n* = 3). Swelling Ratio(%)$=\frac{\mathrm{Ws}\;-\;\mathrm{Wd}}{\mathrm{Wd}}\;\times \;100\%$

$$\mathrm{Water}\;\mathrm{Holding}\;\mathrm{Ratio}(\%)=\frac{\mathrm{Wt}}{W0}\;\times \;100\%$$

(3) Photothermal: PPCS (50–200 μg/mL CuS) irradiated with 808 nm NIR (0.5–2 W/cm^2^, 5 min), monitored by thermal camera. Stability tested by 5 cycles (1.5 W/cm^2^). 

(4) Cu^2+^ Release: PPCS in PBS (37 °C, 200 rpm), sampled at 1–36 h, measured by ICP-OES.

### 4.5. Biocompatibility of the Hydrogels

Hydrogel extracts prepared in DMEM (10 g/100 mL, 37 °C, 24 h). HUVECs and L929 cells (5 × 10^4^/well) cultured with extracts for 24–72 h.

(1) Viability: CCK-8 assay (450 nm).

$$\mathrm{Cell}\;\mathrm{Viability}\;(\%)=\frac{{\mathrm{OD}}_{t\;}-\;{\mathrm{OD}}_{b}}{{\mathrm{OD}}_{c}\;-\;{\mathrm{OD}}_{b}}\;\times \;100\%$$

(2) Live/Dead: Calcein-AM/PI staining, fluorescence microscopy.

(3) Cytoskeleton: HUVECs fixed, stained with AF488-phalloidin/DAPI, imaged by confocal microscopy.

### 4.6. Cell Migration and Tube Formation Assays

(1) Migration: Scratch assay with HUVECs (5 × 10^5^/well), measured at 0/24 h by ImageJ (version number: 1.54k 15 September 2024).

$$\mathrm{Migration}\;\mathrm{Ratio}\;(\%)=\frac{S_{t}}{S_{0}}\times \;100\%$$

(2) Tube Formation: HUVECs (1.6 × 10^5^/mL) on Matrigel, cultured 4 h, quantified by ImageJ.

(3) VEGF/HIF-1α: Measured by ELISA and RT-qPCR (QuantStudio 1 Plus).

### 4.7. VEGF and HIF-1α Expression

HUVECs (1 × 10^4^/well) were cultured with the extract for 72 h. VEGF and HIF-1α levels in the supernatant were detected by ELISA. After 72 h of culture, mRNA was extracted from HUVECs (3 × 10^4^ cells/well), reverse transcribed into cDNA, and analyzed by RT-qPCR (QuantStudio 1 Plus, Thermo Fisher Scientific, Wilmington, DE, USA) for VEGF, HIF-1α, and GAPDH expression. Primer sequences are provided in Supporting Information Table S2.

### 4.8. In Vitro Antibacterial Assays

Hydrogels from the PBS (control), PP, PPC, PPC + NIR, PPS, PPCS, and PPCS + NIR groups were placed in 24-well plates, followed by the addition of 100 μL of *E. coli* or *S. aureus* suspension (10^8^ CFU/mL). NIR-irradiated groups were exposed to an 808 nm laser (1.5 W/cm^2^) for 10 min. Following incubation at 37 °C for 4 h, 100 μL aliquots of bacterial suspension were collected, spread-plated onto Luria–Bertani (LB) agar, incubated overnight at 37 °C, and colony-forming units (CFUs) were enumerated. For ultrastructural analysis, bacterial suspensions were fixed with 2.5% glutaraldehyde, subjected to graded ethanol dehydration, and visualized by scanning electron microscopy (SEM).

### 4.9. Reactive Oxygen Species (ROS) Detection

(1) Hydroxyl Radical (·OH): Immerse the hydrogel in 3 mL methyl violet solution (0.135 g/50 mL water, diluted 100-fold), equilibrate in the dark for 5 min, irradiate with 808 nm NIR (1.5 W/cm^2^) for 10 min, measure absorbance at 580 nm using a UV-visible spectrophotometer.

(2) Singlet Oxygen (^1^O_2_): Immerse the hydrogel in 3 mL DPBF solution (0.135 g/50 mL ethanol, diluted 100-fold), equilibrate in darkness for 5 min, irradiate with NIR for 10 min, and measure absorbance at 420 nm.

### 4.10. In Vivo Wound Healing

Male Sprague-Dawley rats (6–8 weeks of age, body weight 180–220 g) were fasted for 12 h prior to induction. Streptozotocin (STZ; BOAIGANG, S2001, Beijing China) was freshly dissolved in 0.1 M citrate buffer (pH 4.5) at a concentration of 10 mg/mL and administered via a single intraperitoneal injection at a dose of 55 mg/kg body weight. Blood glucose levels were measured from tail vein blood using a calibrated glucometer at 72 h post-injection and again at Day 7. Rats with non-fasting blood glucose levels ≥ 11.1 mmol/L on two consecutive measurements were confirmed as diabetic and included in the study; animals that did not achieve this threshold were excluded. The diabetic state was allowed to stabilize for 7 days following confirmed hyperglycemia, after which full-thickness dorsal skin wounds (diameter: 10 mm, created using a sterile biopsy punch) were made on Day 0 of the wound healing experiment. Seven Groups (each group *n* = 6): PBS (negative control), PP, PPC, PPS, PPCS, PPCS + NIR and Tegaderm™. NIR groups irradiated (808 nm, 1.5 W/cm^2^, 10 min) every 3 days for 14 days. Wound area measured (ImageJ) on days 0, 3, 7, 9, 14. Throughout the study, blood glucose was monitored weekly to confirm sustained hyperglycemia. All procedures were conducted under the approval of the Laboratory Animal Ethics Committee of Xinjiang Medical University (approval number: IACUC-JT-20250311-06).

$$\mathrm{Wound}\;\mathrm{Closure}\;\mathrm{Area}\;(\%)=\frac{S_{0}\;-\;S_{t}}{S_{0}}\;\times \;100\%$$

where S_0_ is the actual wound area measured from the Day 0 photograph for each individual animal, and S_t_ is the wound area at subsequent time points.

### 4.11. Histological and Immunofluorescence Analysis

On days 3, 7, and 14, tissue samples were collected from the wound site and surrounding 0.5 cm area. Hearts, livers, spleens, lungs, and kidneys were harvested from 14-day-old rats. Samples were fixed in 4% paraformaldehyde, embedded in paraffin, and sectioned. H&E and Masson’s trichrome staining were performed. Sections from day 14 were immunofluorescence stained with CD31 (1:100), α-SMA (1:200), Collagen I/III (1:200), and CY3-labeled goat anti-rabbit IgG (1:200). Images were acquired using a histopathology scanner (Panoramic SCAN II, 3DHISTECH, ThreeDimensional−Histological Technologies, Budapest, Hungary).

### 4.12. Statistical Analysis

Data were analyzed using SPSS 21.0 and graphed using Origin 2018. Results are expressed as mean ± standard error. Significant differences were determined by one-way analysis of variance (ANOVA) combined with LSD test: * *p* < 0.05, ** *p* < 0.01, *** *p* < 0.001.

## Data Availability

The data presented in this study are available upon request from the corresponding author.

## Supplementary Materials

The following supporting information can be downloaded at: https://www.mdpi.com/article/10.3390/gels12040291/s1, Figure S1: (a) EDS elemental spectrum of CuS NPs. (b) Zeta potential of CuS NPs; Figure S2: (a) Optical photographs and (b) SEM images of PAM, PP, PPC, PPS, and PPCS hydrogels (scale bar: 100 μm). (c) EDS elemental distribution map of the PPCS hydrogel (scale bar: 100 μm); Figure S3: (a) XPS spectrum of PPCS hydrogel. (b) Modulus variation of PPCS hydrogel at frequencies ranging from 0.1 to 10 Hz (1% strain). (c) Water retention rate of different hydrogels after 72 h. (d) DSC curves of PPC and PPCS hydrogels; Figure S4: Tensile cycle curve of PPCS hydrogel after 20 cycles; Figure S5: (a) Digital images showing deformation and recovery of PPCS hydrogel after five minutes of compression, bending, stretching, and twisting. (b) Digital images demonstrating macroscopic adhesion of PPCS hydrogel to various substrates; Figure S6: (a) Cell viability of HUVECs co-cultured with PP, PPC, PPS, and PPCS hydrogel extracts for 24, 48, and 72 h (n = 3). (b) Cell viability of L929 cells co-cultured for 24, 48, and 72 h (n = 3); Figure S7: Live/dead staining images of HUVECs and L929 cells at 24, 48, and 72 h with different hydrogel extracts(scale bar: 200 μm); Figure S8: H&E staining results of rat heart, liver, spleen, lung, and kidney after treatment with different hydrogels (scale bar: 200 μm); Table S1: The compositions of various hydrogels; Table S2: RT-qPCR primer sequences; Table S3: Antibacterial activity of various commonly used clinical antibiotics [40,41,42,43].

## Abbreviations

The following abbreviations are used in this manuscript:

ECM	Extracellular matrix
NIR	Near Infrared
HUVECs	Human Umbilical Vein Endothelial Cells
α-SMA	α-Smooth Muscle Actin
PTT	Photothermal Therapy
HIF-1α	Hypoxia Inducible Factor-1α
VEGF	Vascular Endothelial Growth Factor
STZ	Streptozotocin
