# Supplementary material for: Multifunctional NIR-Responsive Composite Hydrogel with Combinatorial Antibacterial and Regenerative Properties for Diabetic Wound Healing
Source: Gels. 2026 Mar 31;12(4):291. doi: 10.3390/gels12040291 (PMC13116363; doi:10.3390/gels12040291)
Supplement: Supplementary file 1 [file gels-12-00291-s001.zip › gels-4211451-supplementary.pdf]

Supporting Information

Multifunctional NIR-Responsive Composite Hydrogel with Combinatorial Antibacterial and Regenerative Properties for Diabetic Wound Healing

Authors

Shaokai Ji[†,1], Chao Wang[†,1,], Jie Song[2], Hang shi[1], Donglei Wan[1], Chan Huang[1], Hanzhi Fu[1], Xiaohong Cao[1], Heting Wu[1,3,*], and Jian Yang[1,*]

Affiliations

[1] College of Pharmacy, Xinjiang Medical University, Urumqi, 830017, China.

[2] The First College of Clinical Medicine, Xinjiang Medical University, Urumqi, 830017, China.

[3] Engineering Research Center of Xinjiang and Central Asian Medicine Resources, Ministry of Education, Urumqi 830011, China

Corresponding Author

Heting Wu - College of Pharmacy, Xinjiang Medical University, Urumqi, Xinjiang 830017, China; Email: wuheting@xjmu.edu.cn

Jian Yang - College of Pharmacy, Xinjiang Medical University, Urumqi, Xinjiang 830017, China. Email: yj365@hotmail.com

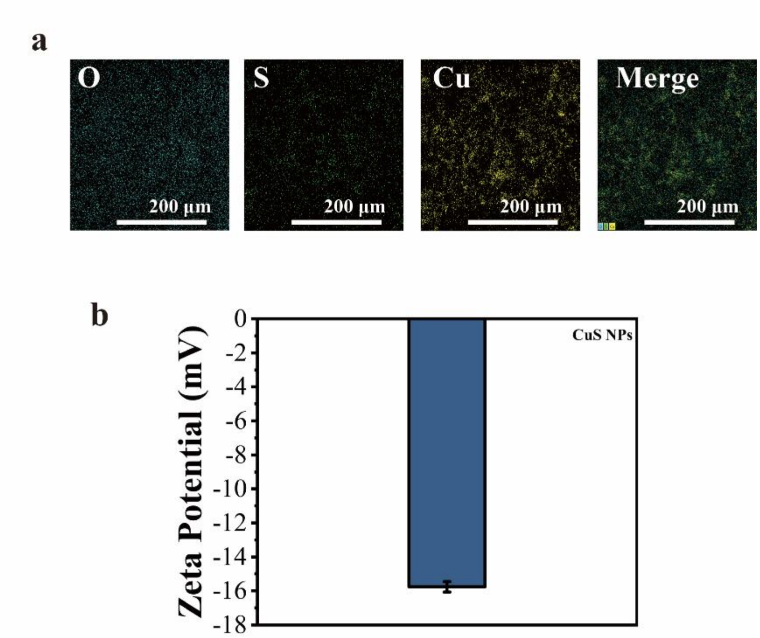

Figure S1. (a) EDS elemental spectrum of CuS NPs. (b) Zeta potential of CuS NPs.

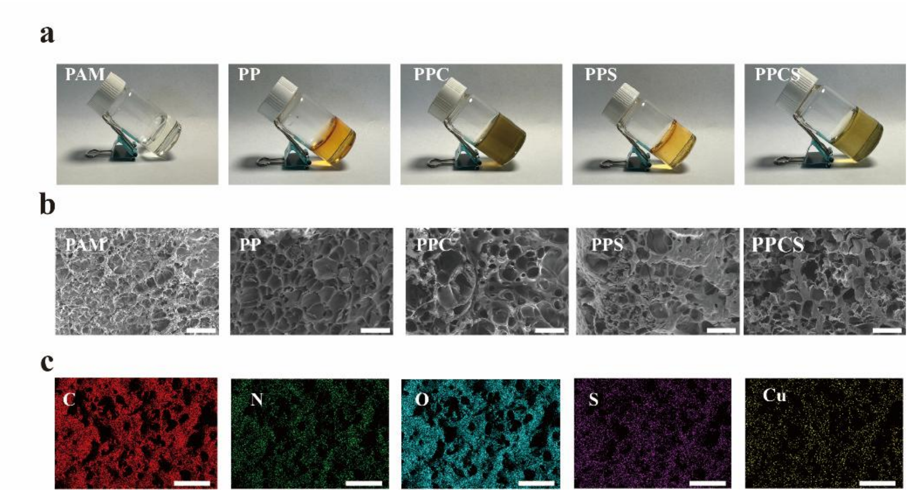

Figure S2. (a) Optical photographs and (b) SEM images of PAM, PP, PPC, PPS, and PPCS hydrogels (scale bar: 100 μm). (c) EDS elemental distribution map of the PPCS hydrogel (scale bar: 100 μm).

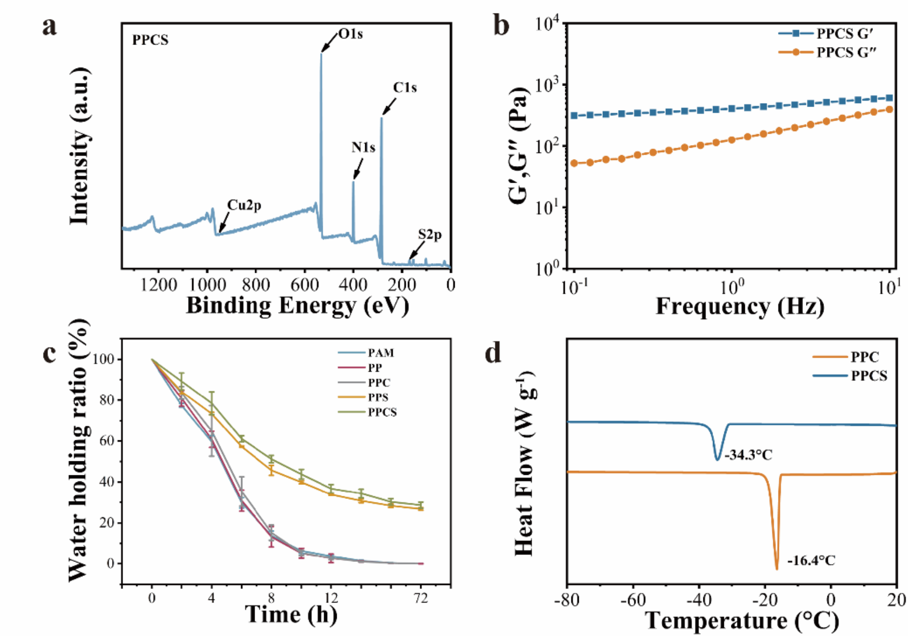

Figure S3. (a) XPS spectrum of PPCS hydrogel. (b) Modulus variation of PPCS hydrogel at frequencies ranging from 0.1 to 10 Hz (1% strain). (c) Water retention rate of different hydrogels after 72 hours. (d) DSC curves of PPC and PPCS hydrogels.

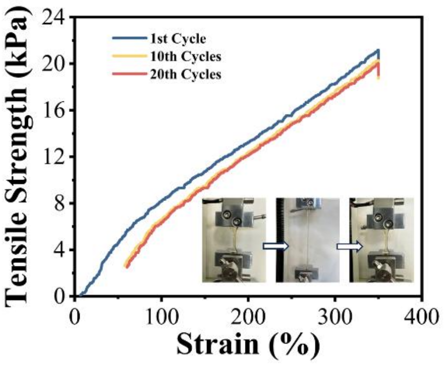

Figure S4. Tensile cycle curve of PPCS hydrogel after 20 cycles.

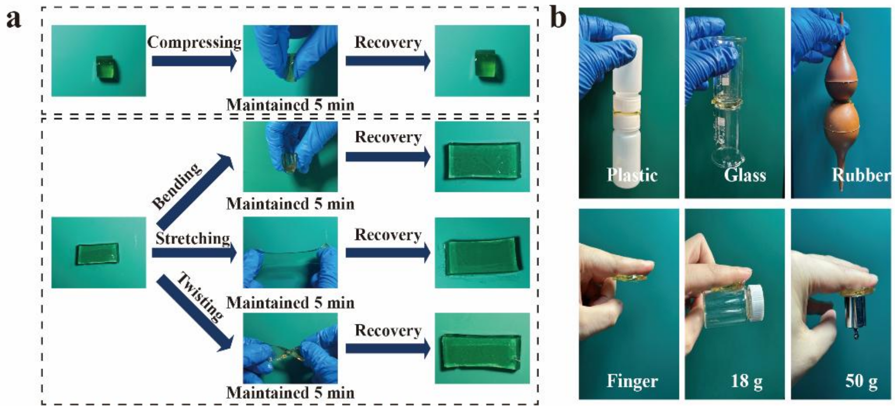

Figure S5. (a) Digital images showing deformation and recovery of PPCS hydrogel after five minutes of compression, bending, stretching, and twisting. (b) Digital images demonstrating macroscopic adhesion of PPCS hydrogel to various substrates.

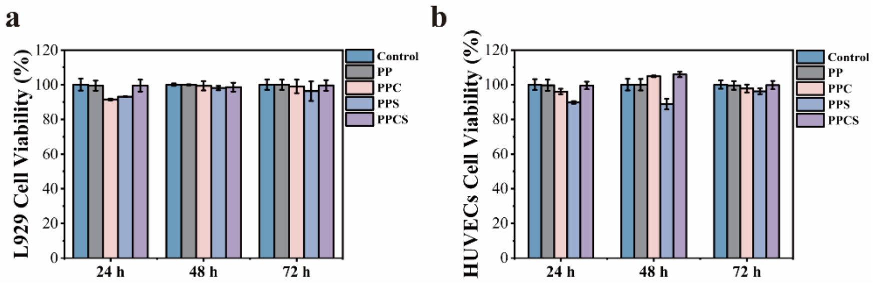

Figure S6. (a) Cell viability of HUVECs co-cultured with PP, PPC, PPS, and PPCS hydrogel extracts for 24, 48, and 72 h (n=3). (b) Cell viability of L929 cells co-cultured for 24, 48, and 72 h (n=3).

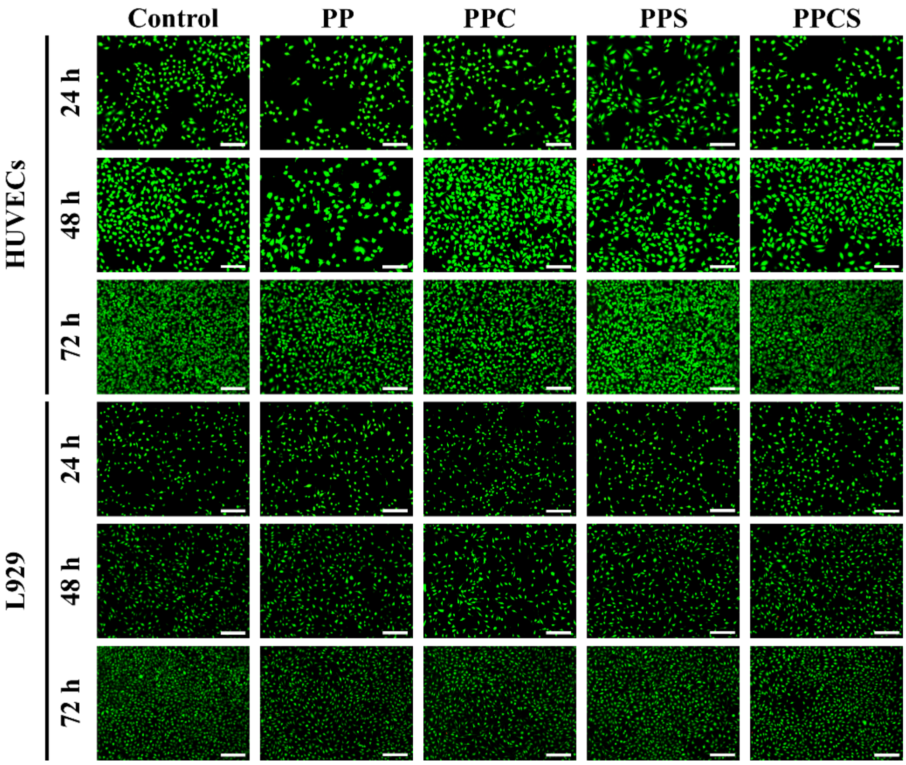

Figure S7. Live/dead staining images of HUVECs and L929 cells at 24, 48, and 72 hours with different hydrogel extracts(scale bar: 200 μm).

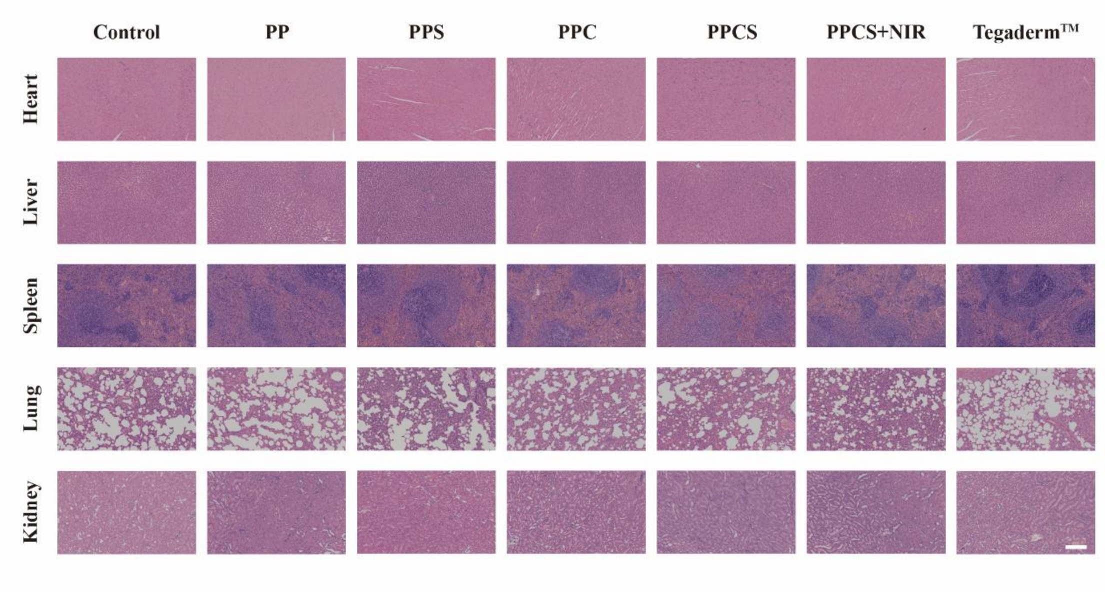

Figure S8. H&E staining results of rat heart, liver, spleen, lung, and kidney after treatment with different hydrogels (scale bar: 200 μm).

Table S1.

The compositions of various hydrogels

Hydrogels	AM (g)	MBA/AM (wt.%)	APS/AM (wt.%)	DA/AM (wt.%)	CuS (mg)	Sucrose (g)	DI Water (mL)
PAM	2.5	1	10	0.5	/	/	15
PP	2.5	1	10	0.5	/	/	15
PPC	2.5	1	10	0.5	0.75/1.5/ 2.25/3	/	15
PPS	2.5	1	10	0.5	/	4.5	15
PPCS	2.5	1	10	0.5	0.75/1.5/ 2.25/3	4.5	15

Table S2.

RT-qPCR primer sequences

Gene name	Primers
GADPH-Forward	TGCACCACCAACTGCTTAGC
GADPH-Reverse	GGCATGGACTGTGGTCATGAG
VEGF-Forward	CCTTGCCTTGCTCTACCTC
VEGF-Reverse	GATGATTCTGCCCTCCTCCTTCTG
HIF-1α-Forward	AGTTACGTTCCTTCGATCAGTTGTC
HIF-1α-Reverse	TCAGTGGTGGCAGTGGTAGTG

Table S3.

Antibacterial activity of various commonly used clinical antibiotics

Name	Antibacterial Ratio% (*S.aureus*)	Antibacterial Ratio% (*E.coli*)	Ref
Mupirocin Ointment	96.3%	/	[40]
Silver-treated medical gauze (30 ppm silver solution)	>99%	95%	[41]
Chlorhexidine Acetate (Proprietary Formula)	⩾90%	⩾90%	[42]
Povidone-iodine solution (containing 1000 mg/L of active iodine)	>99.9%	>99.9%	[43]
PPCS+NIR	99.3%	99.7%	
